# An Unnecessary Pain: Using Pharmacogenetics for Statin-related Skeletal Muscle Toxicity

**DOI:** 10.7759/cureus.2557

**Published:** 2018-04-30

**Authors:** Saeed K Alzghari

**Affiliations:** 1 Gulfstream Genomics, Gulfstream Diagnostics, Dallas, USA

**Keywords:** statins, slco1b1, pharmacogenetics, myopathy, simvastatin

## Abstract

Statins are an important class of medications in reducing the risk of cardiovascular events as well as overall mortality. However, a well-known adverse effect of statins is skeletal muscle toxicity, which may lead to abrupt discontinuation of the statin. In turn, patients may miss out on the benefits of statin therapy. An important factor to consider is a patient's solute carrier organic anion transporter 1B1 (SLCO1B1) gene T521C polymorphism status. Herein, an overview of the pharmacogenetics of SLCO1B1 is provided as well as recommendations for use in practice.

## Editorial

Statins are, by and far, the number one prescribed class of medication worldwide [1]. Their mechanism of action is by inhibiting 3-hydroxy-3-methylglutaryl-coenzyme A (HMG-CoA) reductase, the enzyme responsible for the production of cholesterol. They are effective in lowering low-density lipoprotein (LDL) cholesterol and have consistently shown to reduce the risk of cardiovascular events as well as overall mortality [1].

The most common side effect associated with statins is skeletal muscle toxicity that includes pain without evidence of muscle degradation (myalgias), pain with evidence of muscle degradation (myopathy), and severe muscle damage with acute kidney injury (rhabdomyolysis) [2]. Controlled clinical trials have shown that statin-associated myalgia occurred in 1% to 5% of patients; however, in post-marketing observational cohorts, 11% to 29% of patients experienced statin-associated myalgia [3].

Regarding this association between statins and skeletal muscle toxicity, an important pharmacogenetic marker to consider is the SLCO1B1 gene. The solute carrier organic anion transporter family member 1B1 (SLCO1B1) protein facilitates the hepatic uptake of statins [2]. The most significant variant associated with the SLCO1B1 gene, c.521T>C, leads to decreased transport function of SLCO1B1 that can reduce the clearance of statins. In turn, this could lead to a greater chance of skeletal muscle toxicity. A meta-analysis of nine case-control studies that included 4,442 patients showed that individuals with the variant C allele were significantly more likely to experience statin-related myopathy (TC + CC vs. TT: odds ratio=2.09; 95% CI=1.27-3.43) [4].

The Clinical Pharmacogenetics Implementation Consortium (CPIC) has published dosing recommendations regarding simvastatin and the activity of the SLCO1B1 enzyme (Table 1) [2]. If the normal functioning enzyme of SLCO1B1 is present, then prescribing simvastatin at its normal starting dose is recommended. If a patient presents with the intermediate or low functioning enzyme of SLCO1B1, the recommendation is to consider lowering the simvastatin dose or switching to an alternative statin.

**Table 1:** SLCO1B1 phenotype based on c.521T>C genotype

Phenotype	Frequency of Phenotype in Patients	Definition of Genotype	Genotype of SLCO1B1c.521T>C
Normal function	55-88%	Individual carrying two normal alleles	TT
Intermediate function	11-36%	Individual carrying one normal-function allele plus one decreased-function allele	TC
Low function	0-6%	Individual carrying two decreased-function alleles	CC

CPIC also published information regarding the percent increase in statin exposure in individuals with the SLCO1B1 c.521 CC genotype [2]. Patients with this particular genotype had the greatest statin exposure with simvastatin, the lowest statin exposure with fluvastatin, and other commonly prescribed statins (e.g. atorvastatin, rosuvastatin) fell in the middle (Table 2).

**Table 2:** Percent increases in statin exposure due to SLCO1B1 c.521 CC genotype

Medication (Dose)	Percent Increase in Statin Exposure due to SLCO1B1 c.521 CC Genotype
Simvastatin (40 mg)	221%
Pitavastatin (2 mg)	191%
Pitavastatin (4 mg)	162%
Atorvastatin (20 mg)	144%
Rosuvastatin (40 mg)	117%
Pravastatin (40 mg)	82-99%
Rosuvastatin (10 mg)	65-72%
Fluvastatin (40 mg)	19%

Pharmacogenetic testing for the SLCO1B1 gene can be helpful to clinicians who may have a suspicion that a patient’s statin therapy is causing skeletal muscle toxicity. Also, utilizing SLCO1B1 genotyping can help in deciding which statin may be best for providers initiating statin therapy. Adjustments to the dose of a statin or choosing an alternative can be the difference for a patient especially in those cases where prematurely stopping a statin places the patient at risk of losing the cardiovascular benefits of this therapy. Consideration of following an algorithm where SLCO1B1 gene testing is incorporated in practice can help patients initiating a statin or considering an alternative statin [5]. Providers should be aware of the importance of the SLCO1B1 gene and how it can help guide therapy decisions for their patients.
